# The Landscape of Radiofrequency Technology for Skin Rejuvenation

**DOI:** 10.1002/hsr2.71575

**Published:** 2025-12-27

**Authors:** Boyu Zhang, Xingyu Tan, Qi Zhang, Min Wu

**Affiliations:** ^1^ Department of Plastic and Cosmetic Surgery, Tongji Hospital, Tongji Medical College Huazhong University of Science and Technology Wuhan China

**Keywords:** bipolar radiofrequency, collagen remodeling, monopolar radiofrequency, multipolar radiofrequency, skin aging, skin rejuvenation

## Abstract

**Background and Aims:**

Aging skin is characterized by reduced tissue volume, weakened fibrous support, and increased laxity, leading to wrinkles and sagging. While surgical methods are effective, noninvasive techniques like radiofrequency (RF) have gained popularity due to their safety, minimal recovery time, and natural‐looking results. This review aims to elucidate the mechanisms, classifications, and clinical efficacy of monopolar, bipolar, and multipolar RF systems in skin rejuvenation.

**Methods:**

A systematic review was conducted in electronic databases, including PubMed and Web of Science, to comprehensively analyze the literature on radiofrequency for skin rejuvenation. The methodologies, outcomes, and conclusions of the identified studies were summarized and compared.

**Results:**

RF technology delivers targeted thermal energy to the dermal layer, inducing collagen contraction and stimulating neocollagenesis, thus improving skin texture and elasticity. Monopolar RF achieves deep tissue penetration (up to 20 mm) but requires precise energy modulation to minimize discomfort. Bipolar RF offers localized treatment with reduced penetration depth (1–4 mm), enabling safer home‐use applications. Multipolar RF, particularly microneedle‐based systems, combines mechanical injury with thermal remodeling to enhance collagen production while sparing epidermal integrity. Despite the high safety profile, potential risks and complications exist, emphasizing the need for individualized treatment and experienced practitioners.

**Conclusion:**

Radiofrequency technology is an integral component of skin rejuvenation, whose efficacy depends critically on the customization of treatment parameters and the expertise of the practitioner. Future research should focus on optimizing parameters, elucidating underlying mechanisms, and exploring combination therapies to further enhance clinical efficacy.

**Abbreviations**
FDA: food and drug administration
FEWS: fitzpatrick elastosis wrinkle scale
FLR: facial laxity rating
GAIS: global aesthetic improvement scale
GDWCSS: global drooping and wrinkles classification and scoring system
HVRS: hand volume rating scale
px: pixels
RF: radiofrequency
TEWL: transepidermal water loss

## 1 Introduction

Aging is an inevitable biological process characterized by gradual changes in skin structure and function. As we age, the skin undergoes a series of intrinsic and extrinsic changes, including the loss of collagen and elastin fibers, which are crucial for maintaining the structural integrity of skin. This degradation leads to decreased skin elasticity, the formation of wrinkles, and overall skin laxity [1]. Additionally, factors such as UV exposure, pollution, and lifestyle choices exacerbate these changes, accelerating the aging process. Various treatment modalities have been developed to combat these signs of aging, ranging from topical applications and injectables to more invasive surgical procedures. However, the demand for noninvasive, effective, and safe treatments has significantly increased in recent years, driven by the desire for minimal downtime and reduced risk of complications [2].

**Figure 1:**
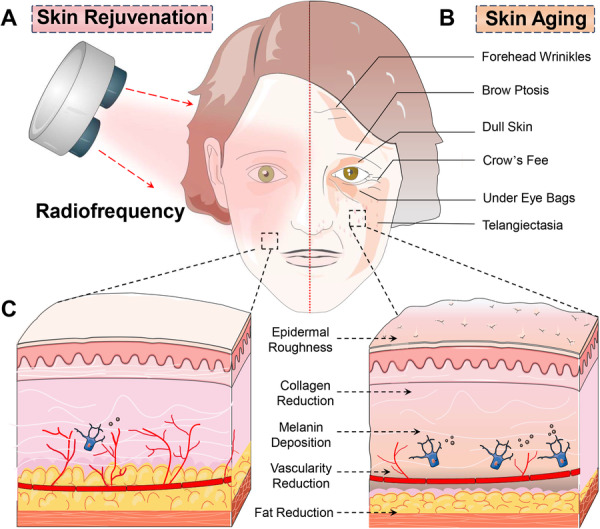
Comparative analysis of the skin phenotype before and after radiofrequency treatment. (A) Posttreatment assessments: Significant reduction in forehead wrinkles and breadth of the forehead, improved definition of eyebrow contours, improved skin brightness and evenness, reduced puffiness around the eyes, and reduced erythema, resulting in a more homogeneous complexion. (B) Pretreatment assessments: Deep forehead wrinkles, blurred eyebrow contours indicating fatigue, dull and uneven skin tone, noticeable circles, significant under‐eye bags, and visible telangiectasia causing skin irregularities. (C) Histopathological evaluations after treatment: Improved skin texture, increased collagen, reduced melanin, improved vascularity, and restored subcutaneous fat.

Among the noninvasive treatments, RF technology has gained considerable attention for its efficacy in skin rejuvenation. RF treatments utilize electromagnetic energy to generate heat within the dermal layers, which stimulates collagen production and remodeling. This process not only improves skin texture and firmness but also addresses multiple signs of aging, such as fine lines, wrinkles, sagging, acne scars, and pigmentary disorders. The ability of RF to induce neocollagenesis makes it a powerful tool in fighting against skin aging (Figure 1) [3]. Furthermore, RF treatments are versatile and can be customized to target specific areas of concern, making them suitable for a wide range of patients with different skin types and aging issues [4]. The noninvasive nature of RF also means that patients can often resume their daily activities immediately after treatment, which is a significant advantage over more invasive procedures.

Technological advancements in RF devices have further enhanced their effectiveness and safety profiles. Modern RF systems offer precise control over energy delivery and depth of penetration, ensuring that the treatment is both effective and safe. Innovations such as fractional RF, which delivers energy in a grid‐like pattern, allow for more targeted treatment with minimal damage to surrounding tissues. Additionally, the combination of RF with other modalities, such as microneedling and laser therapy, has shown promising synergistic effects, leading to improved clinical outcomes and higher patient satisfaction. These advancements have expanded the applications of RF technology, making it a cornerstone in noninvasive antiaging treatments. Furthermore, ongoing research is focusing on optimizing energy delivery, improving device precision, and minimizing side effects to enhance both efficacy and safety.

Despite the promising benefits, comprehensive research is still needed to fully understand the long‐term efficacy and safety of RF treatments. Standardized treatment protocols and a deeper understanding of the molecular mechanisms involved in RF‐induced collagen remodeling are essential for optimizing clinical applications. Therefore, this review aims to synthesize the latest research findings on RF technology and its roles in combating skin aging. We seek to offer valuable insights for clinicians and researchers dedicated to advancing noninvasive skin rejuvenation techniques. Additionally, this review highlights the significance of RF technology in the broader context of antiaging treatments and explores potential future directions for this evolving field. Understanding these aspects will not only enhance the effectiveness of RF treatments but also ensure their safe application, ultimately benefiting a broader patient population.

## 2 Current Categories of Radiofrequency

Presently, RF therapy is highlighted as an innovative nonablative skin rejuvenation treatment. In 2002, the American Food and Drug Administration (FDA) authorized the first RF device to minimize periocular rhytides [5]. The therapeutic application of this technology was later extended to encompass the treatment of facial rhytides in June 2004, and subsequently all rhytides in December 2005 [6]. Since then, varied types of RF devices have been explored and utilized for skin rejuvenation.

Currently, RF can be based on the number of electrodes, which can be divided into monopolar RF, bipolar RF, and multipolar RF. Monopolar RF penetration may cause greater pain than the other types, while bipolar RF penetration is small but more controllable. Furthermore, the number of receptors affected is also reduced. Multipolar RF is constructed using a series of bipolar arrays, with each pair of positive or microneedles and negative polarities forming a circuit of closed‐loop nature.

RF therapies harness electromagnetic radiation in the frequency range of 3 kHz to 300 MHz to generate an alternating current between two electrodes. Most molecules are polar because there is no complete overlap between the positive and negative charges of their chemical bonds [7]. When RF acts on the skin, molecular dipoles rotate within the dielectric polar molecule, resulting in the oscillation of these molecules, which gives rise to the thermal effect on adjacent tissues [7]. The formula for the energy output is described as follows [8].

$$\text{Energy}\left(J\right)=I^{2}\times z\times t,$$

where *I* = current, *z* = impedance, and *t* = time (seconds). Consequently, the thermal energy generated by RF therapy is immensely restricted by the volume of electrical flow and the impedance of the targeted tissue. The order of increasing impedance is as follows: water, nerves, muscles, collagen, and other proteins, and finally, fat. Thus, Fat tissue generates a strengthened electrical heating effect due to the high impedance. Thereby, RF converts electrical energy into thermal energy concentrated in the dermis and results in the contraction of collagen fibril and thermal injury. Collagen starts to denature and coagulate separately when the dermis is heated to 40°C–48°C and 55°C–70°C [7]. The disruption of hydrogen bonding and the alteration of the molecular structure of the triple‐helix collagen molecule contribute to the immediate tissue contraction [9]. Thermal remodeling of collagen occurs at defined thresholds: 85°C (1 ms) or 67°C (3 s), triggering conformationally stable modifications. However, a lower temperature for longer action time, such as 43°C for 3–5 min, is recommended to achieve the remodeling effect of collagen and avoid the risk of skin burns [10]. The injury induces a wound‐healing response that contributes to neocollagenesis and neoelastogenesis, leading to the restructuring of the dermis and skin rejuvenation.

### 2.1 Monopolar Radiofrequency

**Figure 2:**
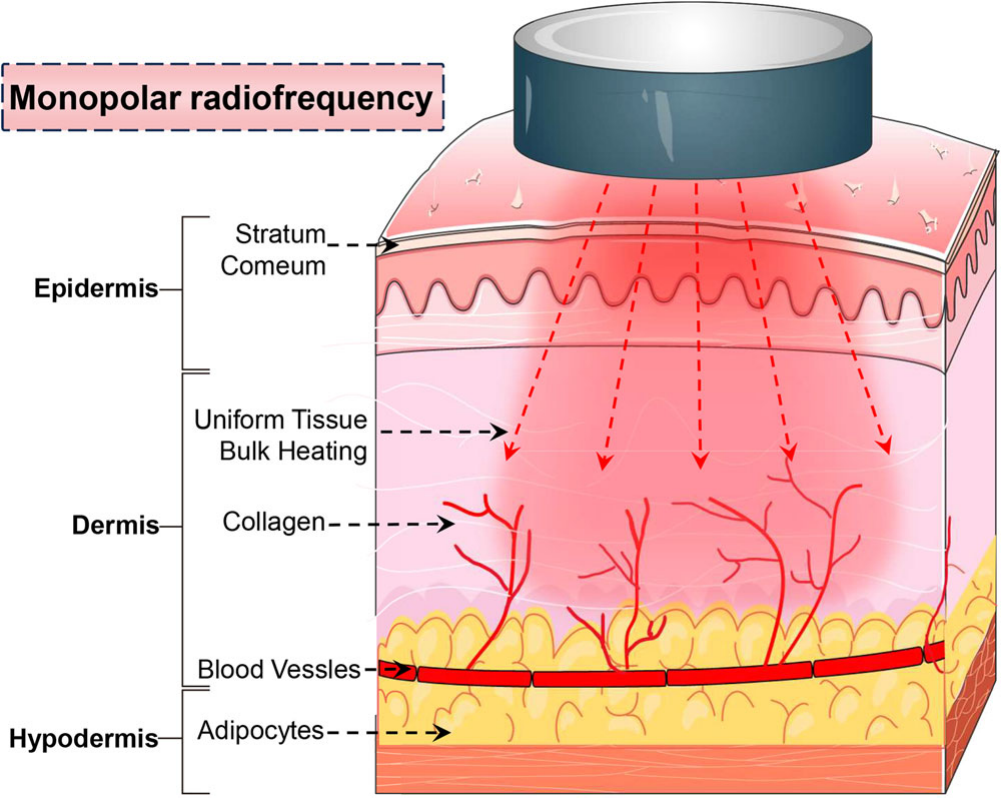
Schematic of monopolar radiofrequency energy delivery. Monopolar radiofrequency energy delivery system comprises a handheld device, which transmits radiofrequency energy to the skin via a single electrode.

Monopolar RF devices comprise an RF generator in combination with a handpiece with an applicator tip [11]. The handpiece is characterized by localized thermal stimulation through a single active electrode, with electric current flowing through the body from the active electrode and out of the passive electrode [10]. The passive electrode is composed of a grounding pad to complete the electrical circuit and is typically located in a region of the body that is relatively away from the active electrode. The main benefit of monopolar RF is that the energy can be dispersed and transmitted uniformly across the surface of the skin, and the penetration of monopolar RF can be up to 20 mm, providing a reliable guarantee for improving skin laxity [12]. However, excessive energy, uncontrollable depth of action, and individual differences in pain perception render the overall comfort of the process dependent on the specialized technologies of the operators (Figure 2).

### 2.2 Bipolar Radiofrequency

**Figure 3:**
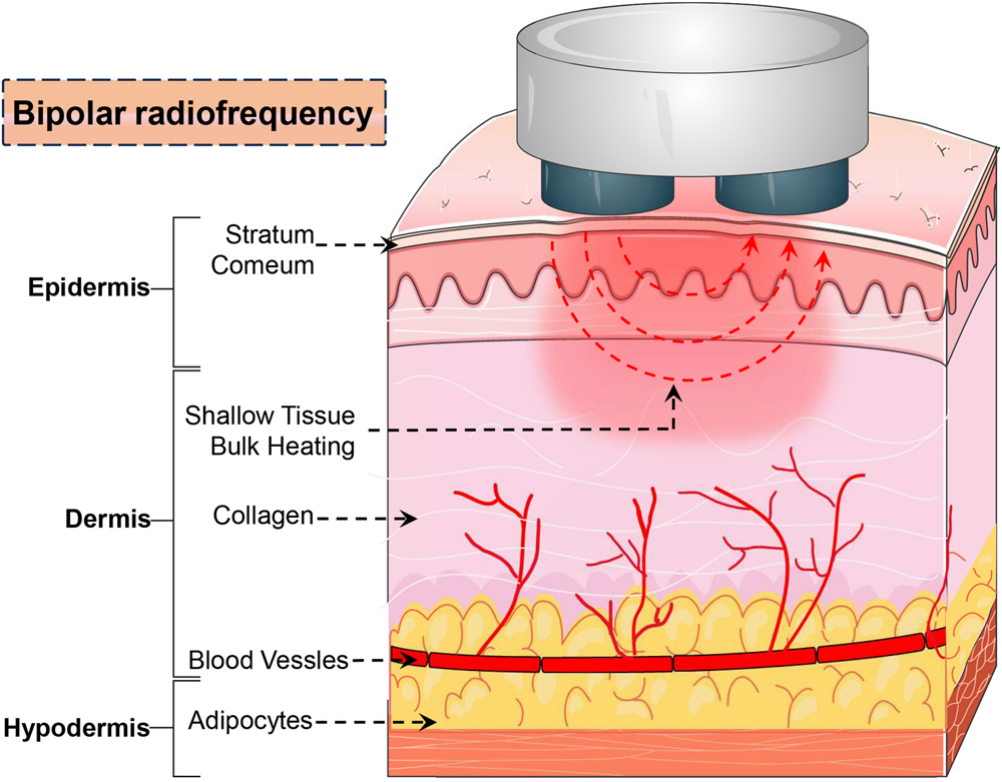
Schematic of bipolar radiofrequency energy delivery. Bipolar radiofrequency energy delivery system comprises two electrodes that function in unison to administer controlled radiofrequency energy to the target region.

Bipolar RF and monopolar RF work on the same principles, but the positive and negative electrodes of bipolar RF are simultaneously located on the surface of the skin without a grounding pad, forming a semicyclic circuit between them, and the theoretical maximum depth of penetration is approximately 50% of the distance between the electrodes [13]. As compared to monopolar RF, bipolar RF provides a more manageable and precise energy distribution pattern, increasing the comfort of the recipients, reducing the difficulties of manipulation, and making the device available for broad application scenarios, such as domestic use [13]. However, this advantage is accompanied by a reduction in the depth of penetration, typically ranging from 1 to 4 mm, as well as a decrease in energy intensity. Nonetheless, the versatility of bipolar RF is evident in its capacity to be integrated with complementary technologies such as fractionation, laser, and multiple electrodes to achieve different penetration depths and the same efficacy as monopolar RF [12]. This hybrid approach not only optimizes the treatment outcomes but also expands the spectrum of clinical indications, thereby enhancing the overall utility and appeal of bipolar RF technology in the field of dermatology and esthetic medicine (Figure 3).

### 2.3 Multipolar Radiofrequency

The multipolar radiofrequency modality utilizes three or more electrodes, with one alternating as the anode while others simultaneously serve as cathodes [14]. Current flow through the active anode equals the cumulative current distributed across all cathodes. Automated electrode polarity rotation prevents localized thermal accumulation at treatment sites by cyclically reassigning anode–cathode roles. The concentrated electromagnetic fields generated through precisely focused current pathways produce elevated therapeutic power densities in target tissues while minimizing systemic energy expenditure. This operational principle enhances treatment durability and reduces procedural discomfort.

**Figure 4:**
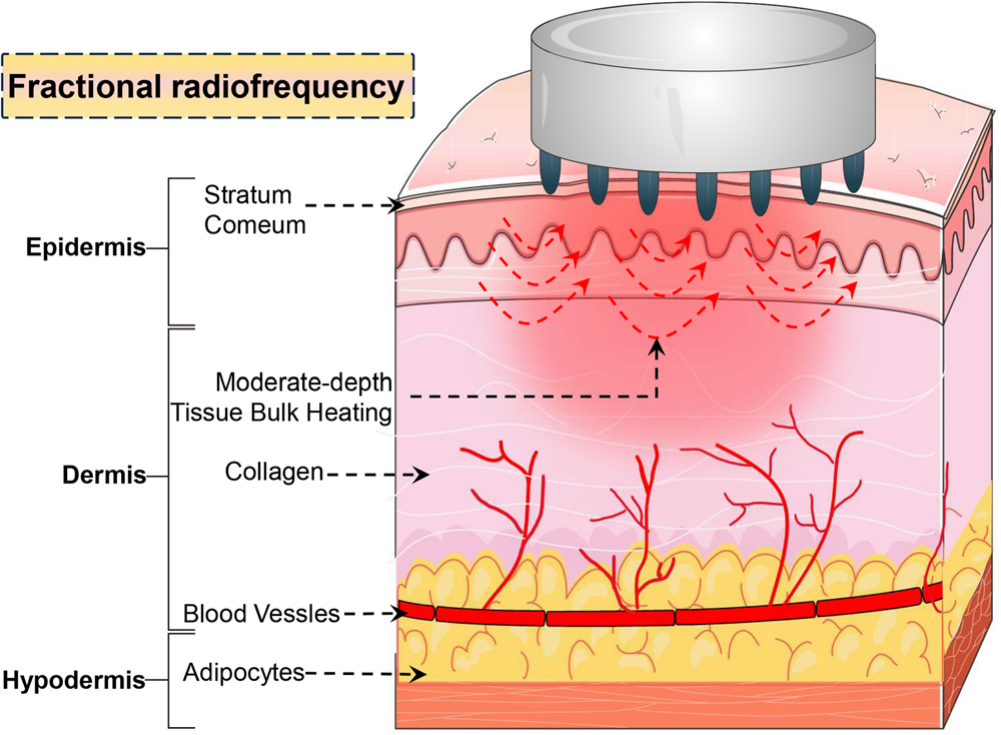
Schematic of fractional radiofrequency energy delivery. Fractional radiofrequency energy is delivered through microneedles or electrode arrays that create microinjuries in the skin to stimulate healing and collagen production.

Fractional RF represents a specialized multipolar configuration wherein electrodes are arranged in an array pattern, achieving enhanced uniformity in energy distribution across the treatment area. Depending on whether it invades the skin, fractional RF is divided into noninvasive and invasive types [5]. Noninvasive fractional RF utilizes electrodes, while invasive fractional RF usually uses a microneedle electrode array to generate current. When fractional RF acts on skin, it delivers thermal energy to the areas where the electrodes or microneedles are located and leaves some unaffected “islands” to serve as the reservoir to accelerate the process of recovery. Hruza et al. reported a kind of noninvasive fractional RF device to investigate its effect on skin [15]. The histological result showed that ablation/coagulation/necrosis zones immediately appeared after the treatment, and more collagen fibers arranged more closely and regularly were seen during the cover time. Hantash et al. first used fractional RF composed of five bipolar microneedle electrode pairs to treat skin in 2009, creating thermal coagulation zones [16]. The depth of energy delivery is contingent upon the design of the microneedle. The transmission of energy along an insulated microneedle occurs solely at the tip of the needle, in contrast to the noninsulated microneedle, which facilitates the transmission of energy over its entire length [17]. The user can control the predetermined treatment layer and temperature with the help of needles. The combination of thermal injury of RF and the mechanical puncture efficacy of needles leads to skin tightening (Figure 4) [15, 18].

## 3 Radiofrequency Technologies in Skin Rejuvenation: Clinical Evidence and Critical Perspectives

Radiofrequency technologies have evolved into cornerstone modalities for noninvasive skin rejuvenation, with monopolar, bipolar, and multipolar systems demonstrating efficacy across diverse anatomical regions and skin types. This section critically evaluates clinical outcomes, mechanistic insights, and practical considerations while synthesizing evidence from landmark studies. By contextualizing findings within broader technological and biological frameworks, we highlight the patterns, contradictions, and opportunities for future research.

### 3.1 Monopolar Radiofrequency: Thermal Precision and Collagen Remodeling

Monopolar RF devices utilize a single electrode to deliver high‐frequency alternating current, generating uniform volumetric heating in the deep dermis and subcutaneous tissue. This thermal stimulation triggers immediate collagen contraction and delayed neocollagenesis, making it particularly effective for skin tightening and contouring.

The multicenter trial by Fitzpatrick et al. established foundational evidence, reporting 83.2% periorbital improvement and 61.5% eyebrow lifting > 0.5 mm in 86 patients using Thermage [19]. These outcomes align with Suh et al. in their histological findings, which showed that collagen fiber density and coherency increased significantly in both papillary and reticular dermis after monopolar RF treatment [9]. Notably, the 49.68% increase in zygomatic length ratio observed by Suh et al. at 4 weeks posttreatment suggests that early collagen contraction precedes long‐term remodeling, which is a critical insight for optimizing treatment intervals [20].

While satisfaction rates ranged from 50% to 82%, disparities in reported outcomes emphasized the influence of technical variables [19, 21]. Hwang et al. noted that 73.5% of patients observed improvements within 2 months [21], whereas Han et al. reported sustained wrinkle reduction (95.08 ± 31.93 mm^2^ to 80.4 ± 28.96 mm^2^) at 1‐month follow‐up [22]. These variations may reflect differences in energy delivery protocols: Thermage FLX (single session) versus Duet RF (three sessions). Importantly, the multicenter study by Labadie et al. highlighted the role of device ergonomics [23]. Hands‐free bipolar RF systems achieved comparable collagen induction while minimizing procedural discomfort.

Additionally, monopolar radiofrequency therapy prevents burns by using surface cooling, but this approach has a certain trade‐off between safety and penetration depth. The absence of standardized protocols across studies complicates cross‐trial comparisons. For instance, the GDWCSS improvements reported by Garg et al. utilized three sessions of Exilis BTL Aesthetics, whereas single‐session protocols dominate newer devices [24]. Future studies should prioritize protocol transparency and histological validation to disentangle technology‐specific effects from operator‐dependent variables.

### 3.2 Bipolar Radiofrequency: Targeted Delivery and Combination Strategies

Bipolar systems employ two electrodes to confine current flow between them, enabling superficial‐to‐mid‐dermal targeting. This precision has expanded applications to pigmentary disorders and scar remodeling, often combined with microneedling or topical agents. Nelson et al. demonstrated graded improvements in 14 patients: 21% substantial, 50% moderate, and 29% slight enhancement via bipolar RF (InMode Inc., Richmond Hill, ON, Canada, North America) [25]. Cutometer data from Kołodziejczak & Rotsztejn corroborated elasticity improvements (R2, R6, R7 parameters) after five sessions, though the lack of long‐term follow‐up (≤ 3 months) limits conclusions about durability [26].

Furthermore, modulation of the dermal microenvironment by bipolar RF has been proven particularly impactful for melasma and other pigmentary conditions, driving significant application expansion in skin rejuvenation and melasma management. Kwon et al. reported a 13.7% melanin index reduction via neocollagenesis and basement membrane restoration [27], while Tsai et al. achieved synergistic effects by combining noninsulated bipolar RF microneedling with cysteamine, a paradigm shift for refractory melasma [28]. However, Gulfan et al. proposed that polynucleotide‐RF combinations offered no superiority over RF alone challenges the assumption that adjuvant therapies universally enhance outcomes [29].

The versatility of bipolar RF is tempered by its limited penetration depth, making it less effective for deep laxity compared to monopolar systems. The bipolar RF exploration by Martin et al. achieved a mean FLR score improvement from 5.6 to 6.3, and highlighted modest efficacy for midface lifting, suggesting a niche role in mild‐to‐moderate aging [30]. Notably, the work on RF microneedling for melasma relapse prevention by Han et al. exemplified how bipolar systems excel in adjuvant roles rather than standalone therapies [31].

### 3.3 Multipolar and Fractional Radiofrequency: Synergy and Innovation

Multipolar RF systems use phase‐shifted currents from multiple electrodes to create overlapping thermal zones, enhancing safety through energy dispersion. Fractional RF further refines this via micro‐insulated needles, enabling precise dermal ablation and collagen remodeling.

Multipolar RF technology induced significant clinical improvement in facial rejuvenation via targeted thermal stimulation, with efficacy corroborated through histological examination. Kim et al. demonstrated universal periorbital improvement using GENIUS fractional RF (Lutronic) with 1.8‐mm depth and 60 mJ/pin energy [32]. Suh et al. expanded on this, showing 53.3% “very much improved” ratings at 4 months posttreatment via Profound system [33]. Histologically, these RF devices, via multiple studies, have been confirmed to trigger a biphasic response, including immediate collagen denaturation followed by neocollagenesis [9, 33, 34].

The capacity of fractional RF to generate controlled focal dermal injury renders fractional RF a viable modality for atrophic acne scars. Sirithanabadeekul et al. reported 38% scar volume reduction posttreatment [35], while Fusano & Bencini confirmed efficacy via confocal microscopy [36]. Paradoxically, Eubanks et al. observed that the configuration of the tip, whether 80‐pin or 160‐pin, had a minimal impact on the outcomes, with GAIS improvements of 1.06 and 0.85, respectively [37]. This indicates that the main factor influencing the clinical outcome is the energy density, rather than the shape of the needle.

While multipolar/fractional RF boasts superior safety profiles, its reliance on multiple sessions raises compliance concerns [34]. Nguyen et al. reported a wide submental volume reduction range (−26.65 to +16.01 cm^3^), emphasizing patient‐specific variability [38]. The authors posit that individualized parameterization could mitigate such disparities, and this assumption needs to be further verified.

## 4 Systematic Comparison of RF Modalities

The studies demonstrated advantages and limitations across RF subtypes, necessitating evidence‐based technology selection (Table 1). Monopolar RF achieved deep dermal penetration through unidirectional current flow, making it optimal for global laxity correction in patients with significant skin redundancy. However, the fuse of monopolar RF energy distribution correlated with higher pain scores compared to bipolar systems. Conversely, bipolar RF confined energy between dual electrodes, enabling precise superficial targeting, ideal for periorbital wrinkles and home‐use devices. While safer for sensitive areas, its limited depth necessitated multiple sessions for sustained collagen remodeling. Multipolar RF bridged this gap by combining fractional ablation via microneedle arrays with RF heating. This modality demonstrated superior efficacy in acne scar revision and photoaging due to spared tissue islands, accelerating healing. However, variability arose from the insulation status of microneedle tips, as noninsulated tips posed a risk of epidermal injury, while insulated tips targeted the deeper reticular dermis.

**Table 1:** Comparative analysis of monopolar, bipolar, and multipolar RF technologies.

Parameter	Monopolar RF	Bipolar RF	Multipolar RF
Penetration depth	Up to 20 mm	1.0–4.0 mm	0.5–3.0 mm
Energy distribution	Diffuse, deep thermal zones	Localized, superficial focus	Microthermal zones with spared areas
Key advantages	Deep dermal remodeling	Precise control, low discomfort	Targeted treatment with minimal downtime
Limitations	Higher pain perception	Limited depth efficacy	Risk of pinpoint bleeding
Clinical indications	Moderate‐severe skin laxity, periorbital wrinkles, facial contouring	Mild‐moderate skin laxity, texture improvement, melasma, home‐use devices	Acne scars, photoaging, enlarged pores,
Typical sessions	1–2 sessions	3–6 sessions	3–4 sessions

## 5 Discussion

RF technology has emerged as a significant noninvasive modality for skin rejuvenation, offering notable advantages in collagen stimulation, skin texture enhancement, and the mitigation of aging signs. This review highlights the diverse applications and efficacy of different RF devices, including monopolar, bipolar, and multipolar RF, in promoting skin rejuvenation.

RF devices deliver targeted thermal energy to deeper skin layers, which facilitates collagen remodeling and neogenesis [39, 40]. This process effectively improves skin elasticity and firmness, making RF a valuable tool in esthetic dermatology. Unlike surgical interventions, RF treatments are less invasive, carry a lower risk, and require minimal recovery time, making them accessible to a broader patient population. Additionally, RF technology is suitable for all skin types, which further enhances its applicability in cosmetic dermatology [41, 42].

Monopolar RF remains pivotal for addressing deep tissue laxity, with studies demonstrating eyebrow elevation (61.5% of patients) and sustained collagen density improvements at 6 months posttreatment. However, its utility is tempered by procedural discomfort and operator‐dependent energy modulation. In contrast, the confined energy delivery of bipolar RF minimizes collateral damage, making it ideal for sensitive regions like the periorbital area. Emerging evidence extends its applications to melasma management, where localized heating reduces senescent fibroblasts and melanin indices by 13.7%, as shown in Korean cohorts. Multipolar RF, particularly microneedle fractional RF, combines the benefits of thermal energy and mechanical injury to achieve significant skin rejuvenation. The use of microneedles allows for precise targeting of the dermal layer, promoting collagen induction and skin tightening with minimal damage to the epidermis. Clinical studies have shown that fractional RF can effectively treat photoaged skin, reduce pore size, and improve skin texture, elasticity, and acne scars. Notably, combination therapies yield superior melasma clearance compared to monotherapy, confirming the value of multimodal approaches.

Despite the widespread clinical acceptance of RF treatments, there is room for further improvement. Enhancements in energy delivery precision and the development of advanced temperature control mechanisms can make RF treatments more effective and safer. Moreover, integrating real‐time monitoring systems to analyze skin conditions during treatment can reduce procedural complexity and enhance patient safety. Future research should also explore the synergistic effects of combining RF with other therapies, such as hyaluronic acid, intense pulsed light, and laser treatments, to achieve superior outcomes [43, 44, 45].

Current studies have several limitations that need to be addressed. Firstly, while the clinical efficacy of RF for skin rejuvenation is well‐documented, the underlying biological mechanisms require further elucidation. Understanding the molecular pathways and changes in gene expression induced by RF can provide insights into optimizing treatment parameters. Secondly, the evaluation methods for RF efficacy need refinement. Incorporating advanced skin imaging technologies, such as reflectance confocal microscopy and optical coherence tomography, can provide more accurate assessments. Additionally, patient‐reported outcomes should be prioritized to capture the subjective experiences of patients undergoing RF treatments. Moreover, the limitations of animal and in vitro models in replicating human skin complexities necessitate more robust clinical studies. Future research should focus on randomized controlled trials with larger sample sizes and extended follow‐up periods to validate the long‐term efficacy and safety of RF treatments. Multicenter studies can also provide more comprehensive data and enhance the generalizability of findings.

As interdisciplinary collaborations advance, it is anticipated that RF technology will continue to evolve, offering improved treatment experiences and outcomes. The integration of novel technologies and combination therapies holds promise for further enhancing the efficacy and safety of RF treatments in esthetic medicine.

## 6 Conclusion

In summary, this review comprehensively concludes the pivotal roles of RF technology in advancing dermatological treatments and optimizing patient outcomes in esthetic medicine. Monopolar, bipolar, and multipolar RF devices demonstrate versatile therapeutic efficacy in addressing a spectrum of cutaneous aging manifestations, including wrinkle attenuation, dermal tightening, acne scar remodeling, and melasma amelioration (Table 2). The high safety profile and minimal downtime associated with RF treatments make them an attractive option for patients seeking noninvasive rejuvenation solutions. It is expected that the understanding of the biological mechanisms of RF and the development of more sophisticated devices will further enhance its clinical applications, providing even better outcomes for patients.

**Table 2:** Studies evaluating the effects of monopolar/bipolar/fractional radiofrequency.

Type of RF	Study design	Results	Ref.
Monopolar	Single‐centered clinical trial	Collagen fiber densities increased to 0.773 ± 0.044 (*p* = 0.018) and 0.686 ± 0.05 (*p* = 0.045) respectively in the papillary and lower reticular dermises	[9]
Monopolar	Prospective, multicentered clinical trial	Fitzpatrick wrinkle score improvement of at least 1 point in 83.2% (99/119) of treated periorbital areas, eyebrows lift of at least 0.5 mm in 61.5% (40/65)	[19]
Monopolar	Single‐centered clinical trial	The average zygomatic length ratio (L1/L2) from the middle third of the face increased by 49.68% in the fourth week (*p* = 0.047), the average perioral perpendicular length reduced to 282.25 ± 84.069 px after 4 weeks, and to 281.38 ± 76.03 px after 24 weeks	[20]
Monopolar	Nonrandomized nonblinded	82% satisfaction with the RF treatment, improvements in skin laxity (52.9%), skin texture (17.6%), and skin tone (11.7%)	[21]
Monopolar	Randomized clinical trial	Wrinkle improvement ratio of 15.19%	[22]
Bipolar	Prospective, multicentered clinical trial	Improvement in skin appearance and an increase in collagen and elastic fibers in the papillary dermis	[23]
Monopolar	Nonrandomized nonblinded	Improvement in all parameters (volume alteration and wrinkles) of the GDWCSS	[24]
Bipolar	Single‐centered clinical trial	21% (3/14) significant improvement, 50% (7/14) moderate improvement, 29% (4/14) mild improvement	[25]
Bipolar	Nonrandomized nonblinded	Improvement in skin elasticity through the result of cutometric measurements of R2, R6, and R7	[26]
Bipolar	Single‐centered clinical trial	The lesional melanin index decreased by 13.7%	[27]
Bipolar	Randomized clinical trial	Significant reduction in melasma severity and enhancement of skin texture	[28]
Bipolar	Randomized clinical trial	Significant improvements in the melanin index	[29]
Bipolar	Retrospective study	Increased average midface laxity score from 5.6 to 6.3	[30]
Bipolar	Randomized clinical trial	Continuous RF therapy is beneficial to maintain conventional melasma treatment outcomes	[31]
Fractional	Nonrandomized nonblinded	Wrinkle improvement of all patients in the lateral periorbital area, improvement in the lower eyelid of two patients, and denser collagen and elastin content in the dermis	[32]
Fractional	Retrospective study	53.3% very much improved result, 26.7% much‐improved result, and 20% improved result of Consensus ratings	[33]
Fractional	Nonrandomized nonblinded	Decreased skin pores, spots, and TEWL (by 18.44%), increased dermal and epidermal density and thickness	[34]
Fractional	Single‐centered clinical trial	A significant decrease in mean acne scar volume and no concomitant rise in mean melanin levels	[35]
Fractional	Single‐centered clinical trial	Significant improvement in the depth of scars	[36]
Fractional	Single‐centered clinical trial	Acne scar improved in 95.5% of subjects	[37]
Fractional	Prospective, intraindividual controlled, single‐center clinical trial	Apparent improvement of aged lower face and neck skin by the assessment of three‐dimensional photographs	[38]

## Author Contributions

**Boyu Zhang:** writing – original draft, writing – review and editing. **Xingyu Tan:** writing – original draft, writing – review and editing. **Qi Zhang:** conceptualization, writing – review and editing. **Min Wu:** conceptualization, project administration, writing – review and editing.

## Conflicts of Interest

The authors declare no conflicts of interest.

## Transparency Statement

The Corresponding author (Qi Zhang, Min Wu) affirms that this manuscript is an honest, accurate, and transparent account of the study being reported; that no important aspects of the study have been omitted; and that any discrepancies from the study as planned (and, if relevant, registered) have been explained.

## Data Availability

The authors have nothing to report.
